# Understanding factors associated with the translation of cardiovascular research: a multinational case study approach

**DOI:** 10.1186/1748-5908-9-47

**Published:** 2014-04-21

**Authors:** Steven Wooding, Stephen R Hanney, Alexandra Pollitt, Jonathan Grant, Martin J Buxton

**Affiliations:** 1 RAND Europe, Westbrook Centre, Milton Road, Cambridge CB4 1YG, UK; 2 Health Economics Research Group (HERG), Brunel University, Uxbridge UB8 3PH, UK

**Keywords:** Research translation, Research impact, Benefits from research, Research returns, Cardiovascular research, Stroke research, Multinational case studies, Payback framework, Pathways towards impact

## Abstract

**Background:**

Funders of health research increasingly seek to understand how best to allocate resources in order to achieve maximum value from their funding. We built an international consortium and developed a multinational case study approach to assess benefits arising from health research. We used that to facilitate analysis of factors in the production of research that might be associated with translating research findings into wider impacts, and the complexities involved.

**Methods:**

We built on the Payback Framework and expanded its application through conducting co-ordinated case studies on the payback from cardiovascular and stroke research in Australia, Canada and the United Kingdom. We selected a stratified random sample of projects from leading medical research funders. We devised a series of innovative steps to: minimize the effect of researcher bias; rate the level of impacts identified in the case studies; and interrogate case study narratives to identify factors that correlated with achieving high or low levels of impact.

**Results:**

Twenty-nine detailed case studies produced many and diverse impacts. Over the 15 to 20 years examined, basic biomedical research has a greater impact than clinical research in terms of academic impacts such as knowledge production and research capacity building. Clinical research has greater levels of wider impact on health policies, practice, and generating health gains. There was no correlation between knowledge production and wider impacts. We identified various factors associated with high impact. Interaction between researchers and practitioners and the public is associated with achieving high academic impact and translation into wider impacts, as is basic research conducted with a clinical focus. Strategic thinking by clinical researchers, in terms of thinking through pathways by which research could potentially be translated into practice, is associated with high wider impact. Finally, we identified the complexity of factors behind research translation that can arise in a single case.

**Conclusions:**

We can systematically assess research impacts and use the findings to promote translation. Research funders can justify funding research of diverse types, but they should not assume academic impacts are proxies for wider impacts. They should encourage researchers to consider pathways towards impact and engage potential research users in research processes.

## Introduction

Research funders have more opportunities for investment in research than they can support. They need to be able to better understand how best to allocate resources to achieve maximum value from their funding, and to justify continued public-funding or charitable support by demonstrating the payback or full range of impacts from the research they fund [1,2]. The ‘science of science’ is a growing field that aims to understand what works in research funding [3,4]. This involves questions such as, what kinds of research, what kinds of researcher, and what settings are most conducive to ensuring success of the full range of biomedical and health research and its translation?

An increasing number of studies from the knowledge translation field identify a range of factors as making an important contribution to encouraging the uptake of evidence [5]. Grimshaw et al. [6] recommend that, in general, systematic reviews rather than single studies be translated. Others, however, focus on the role of researchers disseminating their research, especially when it is applied research [7]. A review of studies analyzing the factors associated with nurses’ uptake of research findings reported that there were too few studies to allow them to draw firm conclusions on the issue of whether engaging in research led to increased uptake of research findings [8]. And Tetroe et al. [9] examined the role of research funding agencies in a range of knowledge translation activities, but claimed little was known about their effectiveness. So, generally, from the perspective of studies of knowledge translation there does not seem to be much analysis of whether translation is influenced by factors associated with how research is conducted.

From the perspective of studies that start with the aim of assessing the impact of research, progress has been made on identifying examples and assessing the level of impacts from health research, but usually in the context of a single country [10-16]. Some of these examples have systematically explored the comparative impact on healthcare of different types of research (basic and clinical) and different modes of funding (projects, fellowships etc). Other studies have started with a series of health policies and systematically identified factors linked to research use in these cases [17]. However, as far as the authors are aware, no previous study started by estimating the impacts from a diverse series of projects and then worked back from that to identify common factors among those projects which have high or low impact. That is what we attempted in the study described here. To analyze systematically the full range of factors that might be associated with the scientific success of research, and its translation, requires a series of steps. We aimed to conduct a suite of case studies to provide a robust assessment of the level of impacts achieved by a range of research projects (conducted in a number of different contexts) together with a description of the processes involved in devising and conducting the research. Key aspects of the impacts identified in the case studies could then be rated to facilitate an analysis of the payback achieved and the factors associated with it.

This study originated as a researcher-driven project. Therefore, it was necessary to identify a medical field and gain the support of research funders. The cardiovascular and stroke field was highly appropriate because it had been the area in which some of the few previous studies of the science of science had been conducted [18], and it was an area in which major health benefits from the application of research-based interventions had been demonstrated [19]. We put together an international consortium of funders and collaborators from Australia, Canada, and the United Kingdom (UK). They included the English Department of Health, the Canadian Institutes of Health Research (CIHR), and a range of medical research charities: the National Heart Foundation of Australia (NHFA) (now called The Heart Foundation [of Australia]); the Heart and Stroke Foundation of Canada (HSFC); the British Heart Foundation (BHF); and UK Stroke Association. Case studies were undertaken by teams in each country, with the UK team also providing overall project leadership and co-ordination.

The resulting Project Retrosight was, therefore, a multinational study that investigated the payback from a diverse range of cardiovascular and stroke research projects, including their translation into policy, products, practice, and health and economic gain. We have made available separate reports on the detailed methods used [20], the full case studies [21] and the policy implications derived from the study [22]. Here, we draw on some of the key methods and findings, and analyze the implications from the study. Furthermore, we subsequently aimed to enhance the contribution that the study could make to the field of implementation science by supplementing the findings of our original study through applying a parallel approach to a single case. So, again, we started with the impact achieved, and aimed to examine thoroughly the complexities that were involved in the translation of even a single body of research into policy and practice. We wanted to uncover the diversity of theories and conceptual discussions that could contribute to an understanding of the complexities of how various features of just one original research project interact and influence the degree of translation achieved.

## Methods

We undertook a sequence of activities drawing initially on existing methods. We adopted successful methodologies used originally to evaluate health services research [10], and further applied to assess the impacts from research funding in diabetes [23], arthritis [11], asthma [16], and health technology assessments [13,14]. But we also developed new methods to expand the analysis. Furthermore, to reduce the dangers of researcher bias we involved, as far as possible, multiple team members in each stage of the project and incorporated various measures to increase transparency and reduce the need for subjective judgements. Here we list the various stages involved in the project, and then describe each in more detail:

1. Selecting a stratified random sample for case studies;

2. Conducting the multinational set of case studies;

3. Rating payback from each case study;

4. Deriving combined impact categories;

5. Identifying factors that might explain variations in impact;

6. Developing policy recommendations.

### Selecting a stratified random sample for case studies

We opted to focus on project grants/grants-in-aid to restrict the variety and scale of funding in our sample. Lists of projects funded by organizations within the collaboration were used to identify a total of 1,347 grants awarded in our chosen period: 1989 to 1993, a period which allowed at least 14 years between the start of the project and the assessment of impacts. These projects provided a population from which to sample our case studies. The time period chosen reflected a balance between the availability of reliable information on projects and the need to allow enough time to have passed for research paybacks to develop.

More so than in those previous assessments of research impact which had adopted a purposive case study selection [11], we wished to increase the likelihood that our findings could be transferable. We therefore identified the number of detailed case studies funding would permit us to conduct, and randomly chose case studies from our population, but stratified the sample by four key characteristics:

1. Type of research (basic or clinical – for the purpose of this project the examples of health services research were included in the clinical category).

2. Country of research (UK, Canada, Australia)

3. Grant size (large or small)

4. An initial estimate of the level of payback (high or low) from the project using the brief approach described below.

Stratifying according to the estimate of payback was thought to be important to ensure we had a balance of levels of payback in the case studies. While the eventual categorization of case studies by the level of payback achieved did not draw on the original estimates, it was thought important to attempt to ensure we studied a portfolio of cases likely to result in different levels of impact. We hoped this would allow us in the eventual analysis to look for factors shared between higher payback case studies, but not with lower impact case studies. To secure an initial estimate of payback we used a web-based survey that asked Principal Investigators (PIs) a series of brief questions about impacts arising from their grants, from the number of papers published to effects on patient or public health. From their answers we made an initial categorization of payback for each grant. We also asked PIs to classify their grants as basic biomedical or clinical research, according to a standard definition we provided (and we subsequently conducted a sensitivity analysis of the basic biomedical-clinical research categorization provided by the PIs). Separately for each country, we conducted the random sample selection from among the project PIs who responded to the survey. For each country we populated a selection matrix using the three key characteristics (types of research, size of grant, and initial estimate of impact). This gave us eight cells for each country and we randomly selected a case from each cell.

The Canadian team was able to conduct an additional four cases, divided between the various categories. As explained below, one additional case study was undertaken in the UK leaving the final total of 29 cases as being 12 Canadian, nine British, and eight Australian. Of the original 28 researchers selected at random from the various cells, 22 agreed to participate, but three of these 22 case studies were not completed.^a^ Finding suitable replacements contributed to an eventual slight imbalance in the set of cases: five of the eight Australian cases were selected from the high impact group, and three from the low impact group. For the UK there were four selected from the high impact and five from the low impact. In Canada there were six in each group. Overall, the balance between basic (15 case studies) and clinical (14) was maintained, as was that between cases initially estimated to have high impact (15) and low impact (14), and between large size (15) and small (14). Nevertheless, in the basic, small, low impact group we ended up with just two studies instead of the intended three, and both of these were from the UK as an additional case was required in this cell because there was no case study from Australia or Canada [22].

### Conducting the multinational set of case studies

We used a case study approach to provide a rich source of relevant material [24]. Such an approach has proved useful when assessing research impacts [25]. We used the Buxton and Hanney Payback Framework to structure our case studies [10]. It has two elements: five payback categories and the payback model. These have been described in previous studies [10,13,26] and the five payback categories are listed in the first column of Table 1. The payback framework provides a common structure for: examining why the PI applied for the research grant; the responses of the funding committees; the research process, including collaborations, use of shared resources, etc; research outputs (e.g., publications); how those outputs influenced subsequent research topics and careers; how the research was subsequently translated into ‘secondary outputs', through influencing clinical policies or product development; and how the research then translated into the final outcomes of improvements in health and broader economic benefits.

**Table 1:** The impacts in each payback category from the 29 cases, with illustrative examples

**Payback category and number of projects achieving some impact**	**Illustrative examples**
Knowledge production: peer-reviewed publications. All 29 projects	A Canadian project on the determinants of increased growth of vascular smooth muscle in spontaneously hypertensive rats produced 16 articles - cited 849 times.
Research targeting and capacity building: Post Graduate research training; career development of PI and team; transfer of skills; informing future studies. All 29 projects	An Australian project on high density lipoprotein (HDL) led to: many collaborations for the PI; advanced the career of the post-doc; new research techniques; further research funding for the group; and better targeting of other groups’ research through increased understanding of HDL. A UK project on the role of coagulation and fibrinolysis in the pathogenesis of recurrent stroke supported the training of 2 PhDs, an MD and development of a patient cohort and control group that formed the basis of a stream of work. It helped the PI establish his research group.
Informing policy and product development: informing a wide range of policies, including clinical guidelines; informing the development of therapeutic products, diagnostic tests, etc. 23 of the 29 projects	An Australian project that created animal models for myocardial dysfunction had contributed to the decision to create a transgenic facility at the research institute, which later informed the development of a commercial facility. Guidelines recommend a treatment pathway for antiphospholipid antibodies based on a Canadian project on warfarin and thrombosis; work on the follow-on studies informed guidelines for warfarin therapy. A UK project on the incidence, severity and recovery of language disorders following right-hemisphere stroke informed: national guidelines, curriculum development of a speech therapy school, and a patient leaflet.
Health and health sector benefits: health gains from improved treatments and public health; more effective use of healthcare resources; increased health equity. 19 of the 29 projects	An Australian project studying the effects of angiotensin converting enzyme (ACE) inhibitors provided part of the international literature used to justify their adoption in the treatment of LV hypertrophy, hypertension, cardiac disease, etc; there have been major health gains from the introduction of ACE inhibitors. A Canadian project on nimodipine binding in cerebral ischemia was part of a stream of work underpinning some of the Canadian Best Practice Recommendations for Stroke. This study, along with many others, led to health gains and cost savings through the administration of tissue plasminogen activator. A UK project analyzing the results of the Heartstart Scotland initiative to introduce automated defibrillators into all Scotland’s ambulances informed not only guidelines but also the policy of ambulance services in Scotland and England. As a result it has made an important contribution to health gains through the increased survival rate following out-of-hospital cardiac arrest.
Broader economic benefits: benefits to the economy such as greater employment, exports, etc., as a result of commercial development informed by the research; contribution to a healthier workforce through a reduction in production lost by mortality and morbidity. 10 of the 29 projects	The commercial transgenic facility developed in Australia as a result of the animal models for myocardial dysfunction described above is now a multi-million dollar business that exports 80% of its services. A Canadian project on coronary lesions and vasoactivity in salmon led on to a body of work that contributed to the literature showing farmed salmon are a safe source of human dietary omega-3 input, thus contributing to sustainable aquaculture. A UK project on fibrillin deficiency in Marfan Syndrome (a condition affecting perhaps 18,000 people in the UK alone) contributed to international research that improved diagnostic tests and informs preventive management that pushed the average age of death higher and most of this health gain is among people of working age; therefore a number of people have been able to remain active in the workforce.

Source: Adapted with permission from Table S.1 of Wooding et al. (2011) [22].

The project received ethical approval from the University Research Ethics Committee of Brunel University.

In each case, we carried out a face-to-face interview with the PI, which was supplemented by interviews with other members of the research team, their collaborators, competitors or practitioners, or policy-makers who used their work. Where available we reviewed funder documents such as applications and end-of-grant reports. We also reviewed PIs’ publications from the grant period.

Alongside the local case study research we undertook bibliometric analysis. Working with the PIs we identified and agreed papers that were directly related to the research undertaken using the grant funding, in addition to those that were indirectly related. We centrally examined those identified papers that were indexed in ‘Web of Science’ and analyzed their citations.

Each country had its own team of case study researchers. To establish consistency across the teams, we held three workshops, provided templates for interview schedules and write ups, and two members of the UK team reviewed all initial drafts. To ensure historical accuracy the case studies were validated by the PIs and underwent external peer review. We attempted to have each case study reviewed by two experts in the field of the case study’s research—one from the same country as the case study and one from a different country. In the end, 24 case studies were double (or in two cases triple) reviewed, and five cases single reviewed. These reviews were used in the next step.

### Rating payback from each case study

We then used the 29 detailed case studies of research grants to identify and understand factors associated with payback. To do this, we needed to determine which of our case studies had high payback and which low, so we could look for factors shared by the high payback cases and not shared with the others. We have previously made progress with quantifying the paybacks recorded in a set of case studies, but we had recognized limitations in our methods [11,13,14]. For this study, we recruited an evaluation panel of nine members (see Acknowledgements) that included a member of the research team from each country and also independent experts. We provided them with tables summarizing the case study narratives and payback, the bibliometric analysis, and the peer reviewers’ comments on the case studies. We also made the full case studies and peer review comments available. We did not provide the panel with information on the PIs’ initial payback estimates, as we wanted an independent quantification of payback based on our case studies.

We asked the panel to provide a relative rating of the payback from each case study in each payback category—that is, five ratings per study: knowledge production; research targeting and capacity building; informing policy or product development; health and health sector benefit; and broader economic benefit. Each rating was on a scale of 0 to 9, where 0 was no payback, 1 was the least payback in the set of case studies and 9 the most: tied ratings were allowed. Each panel member provided an initial set of ratings: these showed a considerable level of agreement. We then brought them together for a two-day workshop in which we discussed the ratings over which there was most disagreement, and then allowed the panel to rate the case studies a second time. The aim of the workshop was to eliminate differences due to differential understanding of the data provided, but to preserve differences that reflected different judgements about the relative value of the paybacks. We carried out a number of tests and analyses of the rating data, which gave us confidence in the robustness of median values that we used subsequently. (For details see the Methodology Report [20]).

### Deriving combined impact categories

We looked for correlations between the various category ratings and used these to simplify the five payback ratings into two summary measures. The results of this analysis led us to combine the first two and the last three payback categories to produce two mutually exclusive groupings. These two combined impact categories were academic impact (encompassing knowledge production, research targeting and capacity building) and wider impact (encompassing informing policy and product development, health and health sector benefit, and broader economic benefit). The two categories, academic and wider impact, were used in the subsequent analysis.

### Identifying factors that might explain variations in impact

To identify factors associated with impact we used our two impact categories—academic and wider—separately. For each, we ordered case studies into three groups—high-, mid-, and low-impact. High- and low-impact groups could then be compared to a series of binary variables representing the presence or absence of each factor in each case study. (We left a mid impact group to ensure that we had a clear distinction between high and low impact cases).

To identify factors associated with impact, we examined the full case studies, coding the presence or absence of factors using NVivo. We identified an initial list of factors from our background knowledge and through discussions at the project and rating workshops. We also added factors that seemed of interest when we reviewed the case studies. We tackled the coding process by working initially with one-half the case studies. Two members of the research team, working independently, took the high-impact cases and looked for common factors. They then compared notes to identify factors both had observed. Two further members of the research team did the same for the low-impact case studies. The research team then came together and all the factors were reviewed and clarified. We then addressed the cases that had not yet been examined, using the finalised list of factors, and revised the analysis of the initial cases in light of the refined list. Finally, each factor was analyzed quantitatively and qualitatively. To carry out quantitative analysis, we looked at the balance of the factor’s occurrence across high, mid, and low academic and wider impact case studies. We used the Fisher Exact test to give us an estimate of the relative strength of the associations versus the likelihood that these were non-random associations between the variables. For qualitative analysis, we assigned each factor to a coder who reviewed all the occurrences of each theme to ensure consistency of interpretation^b^.

### Developing policy recommendations

We presented our initial list of factors associated with impact at an ‘Emerging Findings Workshop’ in London in April 2010 attended by members of the research collaboration and a number of policymakers and evaluators from organizations funding cardiovascular and stroke research and wider health research, from the UK and overseas. The workshop highlighted a number of areas for further analysis and helped refine our thinking. Taking these suggestions into account and drawing on the contextual knowledge of the research team, we developed our observations into policy recommendations.

### Understanding the complexities of implementation

We subsequently built on the detailed work conducted originally. We identified a study that had had considerable impact in a number of categories. We reviewed those various examples of impact, and separated out the various translation pathways through which the diverse impact had been achieved. We then drew on the team’s accumulated knowledge (supplemented by a brief selective review) and identified existing theories and conceptual perspectives that might illuminate the diverse processes involved in this case, and features of the research that might have contributed to its translation.

## Results

### The cases reveal that a significant number and diverse range of impacts arise from the 29 grants studied

The 29 case studies are presented in a full case study report [21]. Table 1 provides a summary of the range and extent of research paybacks associated with the grants, and provides illustrative examples.

### Variations between the impacts from basic biomedical and clinical research

All the grants studied had academic impact. For the combined wider impact categories all clinical studies had some impact, compared to only nine out of 15 basic biomedical case studies. This is a substantial and statistically significant difference. The finding may be confounded by the longer time lags that are typically required for basic biomedical research to achieve wider impacts. Nevertheless, Table 2 clearly indicates that, the basic biomedical research projects have had a greater academic impact and clinical research a greater wider impact over the timescales studied.

**Table 2:** Mean rating by payback category and type of research

**Payback category**	**Basic biomedical research**	**Clinical research**
**Knowledge production**	5.4	4.4
**Benefits for future research and research use**	5.3	4.6
**Informing policy and product development**	2.5	4.9
**Health and health sector benefits**	1.9	4.0
**Broader economic benefits**	1.1	1.2

Each case study was rated for each payback category by all of the 9 raters on a scale of 0-9.

### No correlation between knowledge production and wider impacts

Table 3 shows that overall there is no significant correlation between the payback category ‘knowledge production’ and the three wider categories, ‘informing health policy and product development,’ ‘health and health sector benefits’ and ‘broader economic benefits.’ The data from the table were used to produce the two combined categories of ‘academic impacts’ (i.e. Knowledge production; Research targeting) and ‘wider impacts’ (i.e. Informing policy and product development; Health & health sector benefits; Broader economic benefits).

**Table 3:** The correlations between ratings in each payback category

	**Knowledge production (KP)**	**Research targeting (RT)**	**Informing policy & product development (IPPD)**	**Health & health sector benefits (HHSB)**	**Broader economic benefits (BEB)**
**KP**	***1***				
**RT**	***0.53***	***1***			
**IPPD**	0.13	0.32	***1***		
**HHSB**	0.12	0.39	***0.79***	***1***	
**BEB**	-0.05	0.23	0.46	***0.52***	***1***

Coefficients in ***bold italics*** are significant at a 0.01 level Spearman’s rho and 2-tailed significance; n = 29^c^.

### Factors associated with high and low impacts

The full analysis identifies a number of factors in cardiovascular and stroke research that appear to be associated with higher and lower academic and wider impacts. Here we focus on three of the most clear-cut findings that related to translation of the research into wider impacts (and sometimes there was high academic impact as well). All three provide empirical evidence on issues that are seen as important for the translation of research and a full account can be found in the project’s policy report [22]. Other factors we identified showed a link with achieving high academic impact but not high wider impact, however, it is not thought relevant to describe them here.

### Engagement with practitioners and patients is associated with high academic impacts and high wider impacts

A track record of engagement with practitioners and patients was defined broadly indicating that the PI had interaction with either group in a way relevant to the planning or organization of the research project, or to achieving impacts, and not simply involving patients in trials or studies. Such collaboration with practitioners and patients was more a feature of clinical than of basic biomedical research. Ten out of 14 clinical case studies showed this kind of collaboration, but only 2/15 basic biomedical studies. The correlation between high impact and engagement with practitioners and patients covers both types of impact: academic and wider. All high academic impact case studies on clinical research (6/6) involved engagement with practitioners and patients, compared to 1/4 for low academic impact case studies. Similarly, all high wider impact case studies (5/5) involved engagement with practitioners and patients, compared to 2/4 for low wider impact case studies. Both the basic biomedical research projects with a track record of collaboration with practitioners and patients achieved a high rating for wider impact. Whatever the cause and effect, it is clear from our case studies that some researchers had made considerable efforts to engage with practitioners and patients, and promote the impact of their research. For example, one of the case studies said of the researcher whose research impact was the focus of the study: ‘As a leading clinician in this field, and Medical Advisor and Director to the UK patient and research organizations, X was well placed to provide ‘official', research-informed advice for clinicians and patients’.

### Strategic thinking by clinical researchers is associated with high wider impacts

We defined strategic thinking by clinicians as having occurred when there was evidence in the case study that the research team had thought through the pathways by which research could potentially be translated into practice. This was identified through the analysis of the text in relevant sections of the case studies, such as those where we reported on researchers answers to questions about why they had applied for the research, and what gaps it might fill. There is some evidence that thinking through the translation and application of clinical research is associated with wider impact. All (5/5) high wider impact case studies on clinical research had evidence of strategic thinking by the PI, compared to 1/4 low wider impact case studies on clinical research.

### Basic biomedical research with a clear clinical motivation is associated with high academic impacts and high wider impacts

We considered that clinically motivated basic research occurs when there is evidence in the case study that the research team has a clearly articulated clinical endpoint and/or is explicit in the case study about their clinical motivation. All high academic impact case studies on basic biomedical research (5/5) demonstrated a clinical motivation, compared to 2/6 low academic impact case studies. Similarly, all high wider impact case studies on basic biomedical research (5/5) demonstrated a clinical motivation, compared to 3/6 low academic impact case studies.

For the five high academic and wider impact case studies on basic biomedical research, three of the researchers had a clinical qualification, although they described their research as being basic biomedical; this was confirmed in the sensitivity analysis of the basic biomedical-clinical research definitions that was conducted. In such cases, motivation can be explained by the researchers’ clinical background, as remarked by some of the PIs: ‘The product you see is not just a clinician; it is a clinician with a strong background in the laboratory’.

### Understanding the complexities of implementation

We identified ‘*Heartstart Scotland’ – analysis of the results of a national programme*[21]*,* as a project that had had considerable impact in several categories. The project studied in the case study was itself an evaluation of the results of the Heartstart Scotland initiative to introduce automated external defibrillators (AEDs) into all Scotland’s ambulances. We explored the pattern of translation pathways through which the diverse impacts were achieved, and identified a complex pattern of at least five different ones being recorded for this single piece, or stream, of research. We then identified that there was one, or more, different existing theories or conceptual discussions that we could link to each translation pathway. This is shown on Table 4.

**Table 4:** Understanding the complexities of research implementation: analyzing the translation pathways in the Heartstart Scotland case study

**Examples of impact identified in the Heartstart Scotland evaluation case study [**[21]**]**	**Description of the how the complex pattern of impacts was achieved in the Heartstart Scotland case study: the diverse translation pathways**	**Examples of the variety of existing theories and conceptual discussions that can be linked to the diverse translation pathways**
There was a regular flow of audit and research data from this evaluation of all attempts at defibrillation made by Scottish ambulance crews. The AEDs had originally been introduced through the Heartstart Scotland initiative led by the BHF and that raised funds from members of the public. The evaluation data made an impact on the management decisions of the Heartstart Scotland program in a formative way, and probably had some influence on the decision to renew the AEDs in Scotland from Scottish Office (ie Government) funds. While the main credit for the increased survival following out-of-hospital cardiac arrest should be allocated to Heartstart Scotland program itself, the various decisions that were informed by the evaluation also made an important contribution.	The research was translated through the close involvement of key figures from the Scottish Ambulance Service in all aspects of the research project. Also, the project PI, Prof Stuart Cobbe, chaired the evaluation sub-committee for the Heartstart Scotland program and from 1992 chaired the Professional Advisory Group to the Scottish Ambulance Service. (Cobbe had also played a role in encouraging the adoption of AEDs in the Scottish Ambulance Service in the first place). Furthermore, members of the research team regularly consulted with ambulance crews who had to provide the data for the evaluation.	This case provides a strong example of the benefits of the collaborative approach between health researchers and potential users: such analysis was developed in the 1970s and 80s by Kogan and Henkel [27] and increasingly confirmed in later reviews and analyses [28-32]. This example goes further than just standard collaborative approaches and provides a rarer example of how ‘Linkage and Exchange’ [33] can successfully work in practice. The Linkage and Exchange concept goes beyond collaboration on research agendas, and suggests there should be a continuing involvement by potential users in the research project, which was the case in this example. Within Scotland, Cobbe could be considered a ‘product champion,’ but the wider application of this term is discussed below in relation to adoption outside Scotland.
The evaluation continued with further funding from the Scottish Office and, especially, the BHF. The evaluation demonstrated that benefits from the intervention (ie introduction of defibrillators at a system level) were maintained. Such maintenance of a high level of benefits compares favourably with other examples of the introduction of comparable initiatives that were not accompanied by continuing evaluation research [34]. It was suggested in the case study that the continuing evaluation contributed to the continuing high levels of benefit from the introduction of AEDs.	Several aspects of this case, while not common, provide an interesting example of how the sustained research funding could be linked to the sustainability of the impact of an intervention. A key reason why the same project continued for so long, and established possibly the most significant database on out-of-hospital defibrillation in any country, was the incorporation of the funding for the project into the long-term funding provided for Cobbe through his BHF Chair funding. The continuing links between the research team and the users in the Scottish Ambulance Service, would probably have contributed to the continued benefits from the introduction of AEDs.	In setting an agenda for Implementation research Eccles et al. (2009) set out the importance of sustainability: ‘Within research itself it is important to examine attributes of sustainability (within individuals, teams, and organizations) and to develop methods to examine whether the effects of interventions are sustained over time.’ ([35]: p.5) Sustainability continues to be included in taxonomies of implementation outcomes [36]. The chair funding was very important in this case, and this form of long-term funding is increasingly being shown to assist with the ability to pursue ideas in a way that can contribute either to sustainability of initiatives, or promotion of ideas that might take a long period before they come to fruition [16].
The research almost certainly contributed to the decisions of some ambulance services in the UK and elsewhere to introduce AEDs.	This was probably caused by several factors, including the role of Dr Douglas Chamberlain, a leading national and international figure in the field of resuscitation, who was a pioneer in the use of defibrillators. In his active promotion of AEDs, he frequently drew on the Heartstart Scotland study; Chamberlain believed the study to be a very careful evaluation. The quality and credibility of the study might therefore have been important in the willingness of Chamberlain to draw on it so heavily. Cobbe’s research ranged from basic research, including on the electrophysiology of the heart, through to health services research such as this evaluation. The case study noted other examples where researchers who conducted a wide spectrum of research could sometimes transfer understandings [23].	Various theories of implementation identify the role that can be played by ‘product champions’ in promoting an innovation [37]. This can be seen as going even further than the role of opinion leaders first demonstrated in the healthcare field by Coleman et al. (1966) [38] and elaborated in the description of ‘expert’ opinion leaders by Locock et al. (2001) [39]. While in this case the innovation Chamberlain was promoting was the actual introduction of a system of defibrillators, his activities contributed considerably to the impact made by the Heartstart Scotland evaluation project, because he frequently quoted this evidence when making the case for defibrillators. His willingness to do so seems to have been influenced by the nature of the evaluation research.
The research made a considerable impact on guidelines of many leading and local organizations in the resuscitation field from 1992 onwards. In relation to a range of issues, and in a number of resuscitation-related guidelines and educational and training programmes, papers from the Heartstart Scotland evaluation project were used as the main evidence, or as one of a small number of supporting papers.	The publications and conference presentations from the PI and other team members played a major role in transferring the findings to potential users beyond those directly involved in the project. The team’s dissemination activities were picked up very widely by many organizations. These included ones with whom the research team had links, eg the European Resuscitation Committee, and others with whom the research team appeared to have no direct links. Much of the impact occurred prior to the findings being incorporated into a meta-analysis in 1999 [40], and the specific papers from the research team continued to be cited on guidelines after 1999.	Dissemination of work is increasingly viewed as a major responsibility of researchers: Wilson et al. (2010) reviewed conceptual frameworks designed for use by researchers to guide dissemination and concluded that most applied health research funding agencies expect some effort on the part of researchers to disseminate their findings [7]. The direct impact of this single study raises key issues in the implementation field. Grimshaw et al. 2012 suggest ‘the basic unit of knowledge translation should usually be up-to-date systematic reviews or other syntheses of research findings.’ [6]: p.1 In this case, however, the impact achieved before the publication of a systematic review was an important contribution.
The research made a considerable impact on the meta- analysis published in 1999 on the effectiveness of defibrillator-capable emergency medical services for victims of out-of-hospital cardiac arrest [40].	The Heartstart Scotland database made the biggest contribution to the review: out of 37 papers, Heartstart contributed one third of the patients, more than double next highest [40]. This again illustrates the impact made by the follow-on funding that allowed the project to continue.	This case study demonstrates the complexity of ways in which even a single stream of research might be translated, and that making a major impact on a meta-analysis might be important, but might just be one part of a much broader picture.

## Discussion

It is likely that many of the paybacks identified in Project Retrosight would not have been identified without the structured, case study approach used in this study; an observation that resonates with the diversity of payback identified in earlier studies on arthritis research and on asthma research [11,16]. However, it is significant that a large number and diverse range of paybacks were found from this random stratified sample of 29 case studies across three countries. Various previous studies have identified the (sometimes considerable) impacts from health research, but have used either a purposive case study selection approach [10-12,16] or were randomly selected from a program of research, such as the National Health Service Health Technology Assessment program in the UK, where wider impacts might be expected [13].

The study found, however, that considerably fewer projects resulted in broader economic impacts than had informed health policy or product development. Similarly, it demonstrated the variation between the impacts achieved by different types of research, with basic biomedical research producing higher levels of academic impact and clinical studies resulting in higher levels of wider impact. Before considering some of the policy implications of our study we explore some of the project’s strengths and weaknesses.

### Strengths and limitations

To be able to advance thinking in the area of understanding scientific success and translation required a complex set of steps, a range of methods, and considerable methodological innovation. We took an existing approach for assessing impact that has been applied in various single countries, and applied it in a common way across three countries giving us a larger number of diverse case studies than in most previous studies. Furthermore, the use of the payback framework encouraged consistency across cases and facilitated comparative analysis. Training and quality assurance checks were undertaken to enhance consistency across an international team and these covered both quantitative and qualitative case study material, and we used standard strategies to ensure rigor. We used a two-stage process with a diverse but expert panel of nine to rate the levels of impact by category for each project. All this contributed to providing us with a comparatively large and robust dataset to which we then applied a series of new approaches to make progress in understanding factors associated with high (and low) payback.

The limitations of the analysis include issues common to many projects based on case studies: the number of cases studies was still only 29; despite our efforts we cannot exclude the possibility that there may have been inconsistencies between projects or countries in case study reporting; and despite attempts to address them, there may have been confounders of which we were unaware. With a limited number of case studies some differences between groups of studies reported and discussed here could have arisen by chance. Because of this, we have been deliberately cautious in interpreting our data and have tested the strength of any associations leading to policy observations. We are also aware that the wider research translation literature covers many issues that were outside the scope of this study, given its primary focus on aspects of the production of research that might be relevant for its translation.

### Policy implications

There are policy implications for health research funders of the findings that while both types of research produce a wide range of benefits, within a time period of 15 to 20 years it is likely that basic biomedical research will produce more of the traditional academic impacts, and clinical research will produce more wider impacts, particularly on health policies and health gain. Importantly, we found that the level of knowledge production was not a predictor of wider impacts. While it is important to remember the caveat about the time lags often involved before wider impacts arise, this finding does at the very least mean that any health research funder wishing to demonstrate that it is achieving wider impacts should not use academic impacts as a proxy for that.

From a funding viewpoint, the findings about the benefits from researcher engagement with patients and practitioners are consistent with the claims in the literature that researchers who want their work to have some influence on users are more likely to be successful if they collaborate in some way with them, as described in previous assessments of impact [10,12,13,15,17], and in the theories, reviews, and conceptual discussions described in Table 4[27-33]. This would suggest that research funders should encourage and support clinical researchers who have a record of engaging with practitioners, patients, and managers in the healthcare system or who can demonstrate a clear intention of so doing.

The data about strategic thinking suggest there are benefits when clinical research is undertaken by those able and willing to think about its potential translation into clinical practice or policy. This may have relevance to initiatives to include discussion of potential translation in research applications, and suggests that it may well be appropriate that UK Research Councils, for example, have been encouraging researchers to think strategically and require them to describe potential ‘Pathways to Impact’. Funders should also consider what mechanisms they might be able to use to assist researchers to consider ways to maximize the potential for research to be translated. But the findings also highlight that research funders and governments more generally should realize that most biomedical and clinical projects are unlikely to produce significant economic benefits within a period of 15 to 20 years. Perhaps along similar lines, the findings about basic research with a clinical motivation being associated with high impact, at least in the time period over which this study was conducted, are findings that funders should consider in relation to their portfolios of research and the timescales over which they hope to make an impact. This also correlates with a previous study of research on diabetes [23].

The full policy implications are set out in the project’s policy report [22], but this selection of key points underlines the significant contribution that ‘science of science’ studies can make to providing an evidence-base to better inform the decisions to allocate funding that health research funders have to make. It would be helpful to see such analysis explored in further case studies and in other areas of health and related research and therefore potentially strengthen the validity of the findings, and develop further the evidence-base for research-funding policy. Furthermore, our findings on Table 4 provide a powerful lesson in the complexity of achieving, and hence also studying, research implementation. They identify the range of impacts that can come from a single case, the diverse translation pathways involved, and the extent to which these pathways can be conceptualised and associated with the nature of the research itself and how it was conducted. This reinforces the view that further work in this field would be beneficial so as to develop a fuller understanding of the diverse and interacting processes that contribute to research translation.

## Conclusions

Previous studies of knowledge translation, and of research impact, tend not to focus on the aspects of research production that might contribute to research implementation. Yet research funders face more opportunities for investment in research than they can support. They need to be better able to understand how best to allocate resources to achieve maximum value. We built on the Payback Framework and expanded its application through conducting a multinational study consisting of 29 co-ordinated case studies on the payback from cardiovascular and stroke research in Australia, Canada, and the UK. Then we devised a series of innovative steps to first rate the level of impacts identified in the case studies, and second to interrogate the full case study narratives to identify factors that correlated with achieving high or low levels of impact.

We found that over the 15 to 20 years examined, basic biomedical research has a greater impact than clinical research in terms of academic impacts such as knowledge production and research capacity building, but clinical research has the greater impact on health policies, practice, and generating health gains. There is no correlation between knowledge production and wider impacts. We identified a range of factors associated with high impact, at least within the timescales considered. Interaction between the researcher and practitioners and the public is associated with high academic and wider impacts, as is basic research with a clear clinical motivation. Strategic thinking by clinical researchers is associated with high wider impact.

Research funders can justify funding research of diverse types, but should not assume academic impacts are a proxy for wider impacts. They should encourage researchers to engage potential research users in the research process, and do what they can to assist researchers consider pathways towards impact. They should also, where relevant, consider the finding that basic research proposals that have a clear clinical motivation seem, at least in a 15 to 20 year timescale, more likely to make a wider impact. Finally, the complexities illustrate that, while health research is clearly shown to be making a considerable impact, there is still much to learn in this field about the most productive combination of approaches to use to enhance the chances of maximizing the impact.

## Endnotes

^a^In one of the three cases, it transpired that the grant did not meet our inclusion criteria, in another study insufficient evidence was available to allow full completion, and in the third the researcher had moved to Japan [22].

^b^We eventually decided not to attempt any analysis based on grant size as the range of sizes turned out to be relatively small, differed between countries and was not always correctly reported in funders’ records [22]. Similarly, we do not include any country comparisons because of concerns that any differences between scores for countries might have been a reflection of the fact that each country had its own specific team to conduct the cases, and despite our attempts to ensure consistency in the conduct of the cases, some differences might have emerged.

^c^We checked two statistical approaches: our initial approach of using all 261 ratings, as described in the report [22]; and a possible alternative of using the 29 median values to calculate the r values. In Table 3 we present the findings from using the latter approach. We found that in terms of the conclusion we draw-i.e., the grouping of the categories-the method used for the test does not affect the conclusion. Using all the 261 ratings ensures we don’t lose data, but probably overestimates the n because they are not fully independent observations; but using the medians may overestimate the magnitude of the correlation because it excludes the outlying observations.

## Abbreviations

ACE: Angiotensin converting enzyme; AED: Automated external defibrillators; BHF: British Heart Foundation; CIHR: Canadian Institutes of Health Research; HDL: High density lipoprotein; HERG: Health Economics Research Group; HSFC: Heart and Stroke Foundation of Canada; NHFA: National Heart Foundation of Australia; PI: Principal Investigator.

## Competing interests

The authors declare that they have no competing interests.

## Authors’ contributions

SW participated in the design of the study, led the data collection and analysis, and helped to draft the manuscript. SH participated in the design of the study and data collection and analysis, and led on drafting the manuscript. AP participated in the data collection and analysis, led the production of the case study report, and helped to draft the article. JG co-conceived the study, participated in the design of the study, and in the data collection and analysis, and helped to draft the manuscript. MB co- conceived of the study, participated in the design of the study and in the analysis, and helped to draft the manuscript. All authors read and approved the final manuscript.
